# Carbon Dot-Assisted Hydrothermal Synthesis of Copper Doped Tin Disulfide Nanosheets for Optoelectronic Applications

**DOI:** 10.3390/ma19112275

**Published:** 2026-05-27

**Authors:** Huijuan Geng, Xiwei Zhang, Shuowei Liu, Mengya Wu, Zhenjie Tang, Yanjie Su, Jiang Zhao

**Affiliations:** 1 School of Physics and Electrical Engineering, Anyang Normal University, Anyang 455000, China; ghjhou@aynu.edu.cn (H.G.); xwezhang@outlook.com (X.Z.); uoihas@163.com (S.L.); w15993995108@icloud.com (M.W.); 2 National Key Laboratory of Advanced Micro and Nano Manufacture Technology, Shanghai Jiao Tong University, Shanghai 200240, China; 3 School of Integrated Circuits (School of Information Science and Electronic Engineering), Shanghai Jiao Tong University, Shanghai 200240, China; 4 College of Integrated Circuit Science and Engineering, Nanjing University of Posts and Telecommunications, Nanjing 210023, China

**Keywords:** SnS_2_ nanosheets, carbon dots, hydrothermal synthesis, optoelectronic properties

## Abstract

Tin disulfide (SnS_2_) has attracted extensive research attention due to its superior properties originating from its unique crystalline structure. However, its practical applications are greatly restricted by difficult morphology regulation and insufficient photoresponse capability. Herein, we successfully synthesized copper and carbon co-doped SnS_2_ (Cu-C-SnS_2_) nanosheets via a carbon dot-assisted hydrothermal method. The morphology, crystal structure and chemical composition of the obtained samples were characterized by FE-SEM, XRD and XPS. The experimental results reveal that the synthesized Cu-C-SnS_2_ presents nanosheet morphology with a bandgap of approximately 2.445 eV. Moreover, carbon dots and copper doping can effectively regulate the morphology of SnS_2_, which provides a reliable strategy for the controllable synthesis of SnS_2_ nanosheets. Meanwhile, the photoelectric device based on the as-fabricated Cu-C-SnS_2_ nanosheets were successfully fabricated, and exhibited favorable photoelectric response under 405 nm light irradiation.

## 1. Introduction

Tin disulfide (SnS_2_), as an important member of IV–VI semiconductor materials, is a transition metal dichalcogenide with a moderate bandgap varying from 2.18 to 2.44 eV [1,2]. SnS_2_ is confirmed to be an n-type semiconductor with a typical CdI_2_-type layered structure [3,4]. A tin atom is found to be enclosed between two layers of closely packed sulfur atoms, by means of which hexagonal stacking arrangements are formed. Strong covalent bonding is established between Sn and S atoms within every individual triple layer, while these separate triple layers are observed to be interconnected through van der Waals forces [5,6,7]. A variety of excellent optical, electrical and optoelectronic properties have been demonstrated for SnS_2_ [8,9,10,11,12,13]. Additionally, it is highlighted that SnS_2_ is abundant, highly stable, low cost, and environmentally benign with low toxicity. Extensive applications of SnS_2_ have been identified in a variety of fields, such as optoelectronic devices [5,14,15,16], lithium ion batteries [17,18,19,20], supercapacitors [21,22,23], photocatalysis [2,24,25,26,27], and so on.

Because of the above wide application, numerous research endeavors have been devoted to the fabrication of SnS_2_ nanomaterials. To date, various morphologies of SnS_2_, including flower-like structures [22,28], nanowires [29], nanobelts [30,31], nanorods [32], nanosheets [3,33], nanoparticles [34], and nanodots [35], have been successfully prepared by various techniques, such as chemical vapor deposition (CVD) [36], solvothermal methods [37], thermal decomposition [38], microwave-assistant methods [23], template methods [39], and so forth.

To investigate the electronic and optoelectronic properties of SnS_2_ nanosheets, systematic doping investigations have been conducted by different research teams. For instance, Na-doped SnS_2_ thin films have been prepared using a simple solution-based method, and it is found that Na significantly improves the photodetection performance of the n-SnS_2_/p-Si heterojunction devices [16]. Zhao et al. also investigated the outstanding photocatalytic performance of Cr-doped SnS_2_ nanoflowers in the dye’s decomposition under visible light irradiation [40]. Zhao et al. successfully synthesized Ga/W co-doped SnS_2_ microspheres and systematically evaluated their gas sensing properties [41]. Tang et al. synthesized Cu-doped SnS_2_ nanosheet/RGO composites, and the results show that these composites possess great potential as high-efficiency visible-light-responsive photocatalysts [26].

In this work, we successfully synthesize Cu-C-SnS_2_ nanosheets using a carbon dot (CD)-assisted hydrothermal method. The effects of Cu doping and CDs on the synthesis and morphological evolution of SnS_2_ nanosheets were systematically investigated. Moreover, the photoelectric devices based on Cu-C-SnS_2_ nanosheets exhibit excellent photoelectric response under 405 nm light irradiation.

## 2. Experimental Section

### 2.1. Methods

Cu-C-SnS_2_ nanosheets have been successfully fabricated via the hydrothermal route with the assistance of CDs. According to a typical process, 0.05 mmol CuCl_2_·2H_2_O, 0.5 mmol SnCl_4_·5H_2_O and 2.5 mmol L-cysteine were dissolved in distilled water (30 mL) under continuous stirring at room temperature. All reagents were purchased from Sinopharm Chemical Reagent Co., Ltd. (Shanghai, China). Then, the CD solution (0~5 mL), prepared in our group [42], was incorporated into the mixture, followed by continuous stirring for an additional 2 h to achieve a well-distributed precursor solution. The as-obtained uniform solution was then transferred into a 50 mL stainless steel autoclave lined with Teflon, which is hermetically sealed and thermally maintained at 180 °C for 24 h before being naturally cooled to room temperature. Ultimately, the black precipitate was collected by centrifugation, rinsed multiple times with deionized water, and dried under vacuum conditions at a temperature of 70 °C.

### 2.2. Characterizations

Characterization of the crystal structure for the as-prepared samples was performed via X-ray powder diffraction (XRD) analysis, employing an 18 kW high-performance X-ray diffractometer (D8 ADVANCE, Bruker, Karlsruhe, Germany) operated with Cu-Kα radiation (λ = 0.154056 nm). Observation of the micro-structural morphologies was implemented by means of scanning electron microscopy (SEM, Zeiss Ultra 55, Oberkochen, Germany). The absorption spectra were recorded using a UV-vis-NIR spectrophotometer (Perkin-Elmer Lambda 750, Shelton, CT, USA). X-ray photoelectron spectroscopy (XPS) data was performed on a Kratos Axis UltraDLD system (Manchester, UK) with a monochromatic Al Kα source, under ultra-high vacuum conditions with a base pressure maintained at 5 × 10^−10^ Torr. The photoelectric properties of Cu-C-SnS_2_ nanosheets were investigated by monitoring I–V curves with and without light irradiation on the prototype photodetector device, which was fabricated by drop casting a dispersion of a SiO_2_ substrate between two Au electrodes with a spacing of 10 μm.

## 3. Results and Discussion

**Figure 1:**
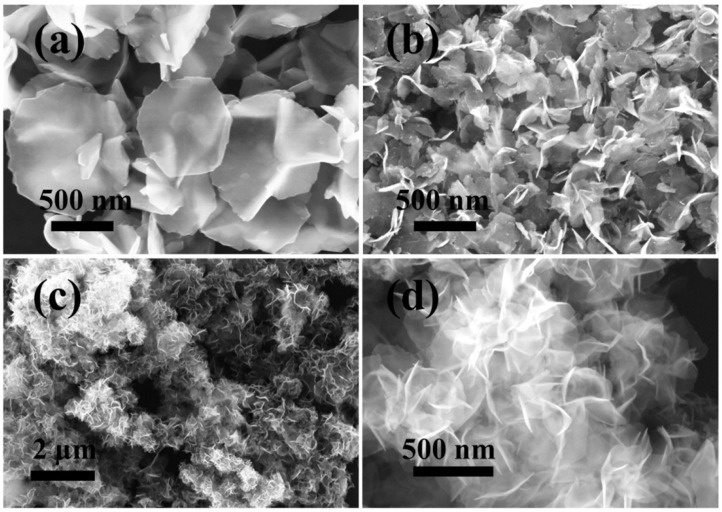
SEM micrographs of the nanomaterial samples: (**a**) SnS_2_; (**b**) Cu-SnS_2_; (**c**,**d**) Cu-C-SnS_2_.

The morphological and structural characteristics of the as-prepared samples are examined by means of SEM. Figure 1 presents the SEM images of the as-prepared SnS_2_, Cu-doped SnS_2_ nanosheets, and Cu-C-SnS_2_ nanosheets synthesized using CD-assisted hydrothermal method.

As observed in Figure 1a, the as-synthesized SnS_2_ samples have a dispersed plate-like nanostructure. Most plates exhibit a typical hexagonal morphology with a size of approximately 600 nm. However, with Cu doping, the morphologies become smaller, and some nanoparticles appear, as shown in Figure 1b. Figure 1c shows a typical morphology of Cu-C-SnS_2_ nanosheets, which is smaller and thinner. A magnified view (Figure 1d) shows that these Cu-C-SnS_2_ nanosheets are thin. This demonstrates that Cu doping coupled with CDs can effectively tailor the nanosheet morphology, yielding thin structures.

**Figure 2:**
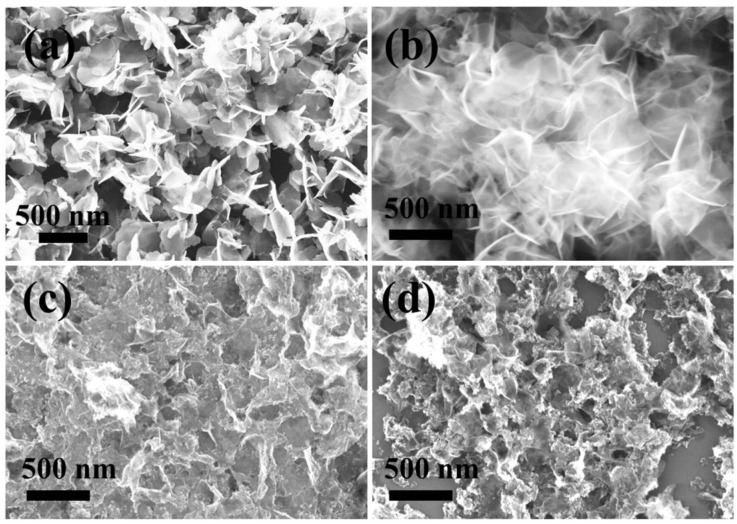
SEM micrographs of Cu-C-SnS_2_ nanomaterial samples obtained with different contents of CuCl_2_·2H_2_O: (**a**) 0 mg, (**b**) 10 mg, (**c**) 20 mg, and (**d**) 40 mg.

The influence of CuCl_2_·2H_2_O dosage on the morphology of SnS_2_ is further investigated in this study. SEM images of the samples prepared with 0 mg, 10 mg, 20 mg and 40 mg CuCl_2_·2H_2_O are displayed in Figure 2.

As revealed in Figure 2a, irregular morphology is observed for the samples synthesized in the absence of CuCl_2_·2H_2_O. With the addition of 10 mg CuCl_2_·2H_2_O, the sample morphology is gradually optimized, and thinner nanoflakes are obtained. These results confirm that the introduction of CuCl_2_·2H_2_O can contribute effectively to the morphological improvement of the samples. Nevertheless, when the CuCl_2_·2H_2_O content is increased to 20 mg and 40 mg, SnS_2_ is transformed into thin films without obvious nanoflake structures. Such morphological evolution indicates that CuCl_2_·2H_2_O plays a key role in the morphological regulation of the samples. And 10 mg of CuCl_2_·2H_2_O is determined as the optimal doping dosage.

**Figure 3:**
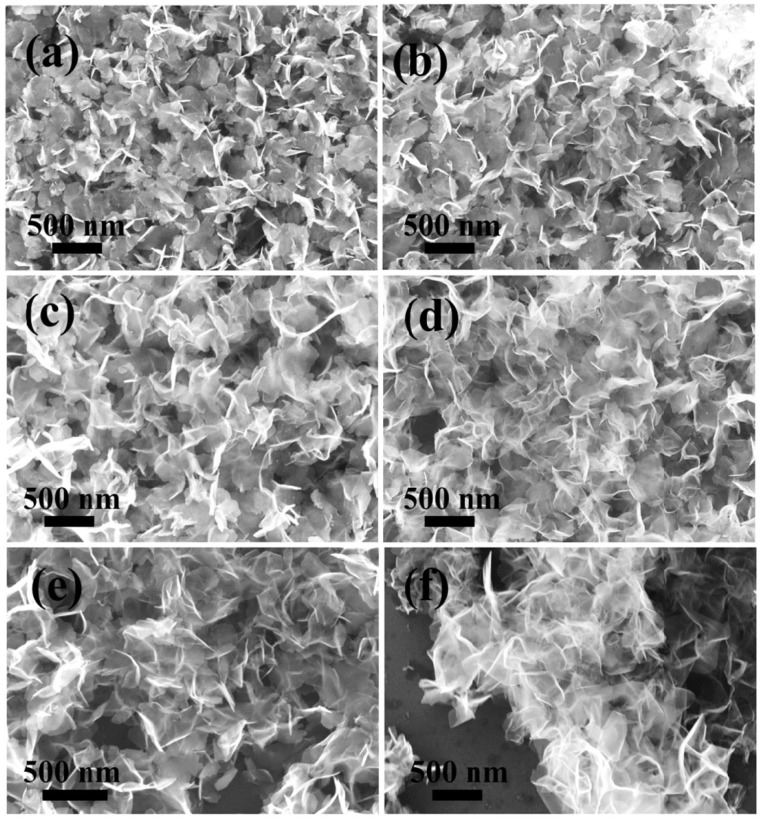
SEM micrographs of the samples synthesized with different volumes of CDs: (**a**) 0 mL, (**b**) 1 mL, (**c**) 2 mL, (**d**) 3 mL, (**e**) 4 mL, and (**f**) 5 mL.

The effect of the amounts of CDs is also discussed, with the corresponding results presented in Figure 3.

As displayed in Figure 3, the addition of CDs facilitates the formation of thin nanosheet structures. When the CD volume reaches 3 mL, the as-prepared Cu-C-SnS_2_ nanosheets become significantly thinner. No obvious morphological changes are observed with further increasing CD dosage.

As reported in previous studies, L-cysteine has three functional groups, including -NH_2_, -COOH and -SH, which can strongly coordinate with inorganic cations and metals [24]. The possible mechanism of this work is proposed as follows: L-cysteine reacts with SnCl_4_ to form plate-like SnS_2_ nanostructures. When SnCl_4_ and CuCl_2_ are added simultaneously, metal–cysteine complex precursors are generated at the initial stage. Due to the moderate reducibility of L-cysteine, Cu^2+^ is reduced to Cu^+^, which gradually optimizes the material morphology. After CDs are introduced, their surface hydroxyl, carboxyl and amino groups exhibit strong affinity with uncoordinated groups of cysteine. Such interaction regulates precursor growth and ultimately forms thin nanosheets. This evolution process is similar to that reported for Cu-doped SnS_2_ nanosheet–RGO composites [26].

**Figure 4:**
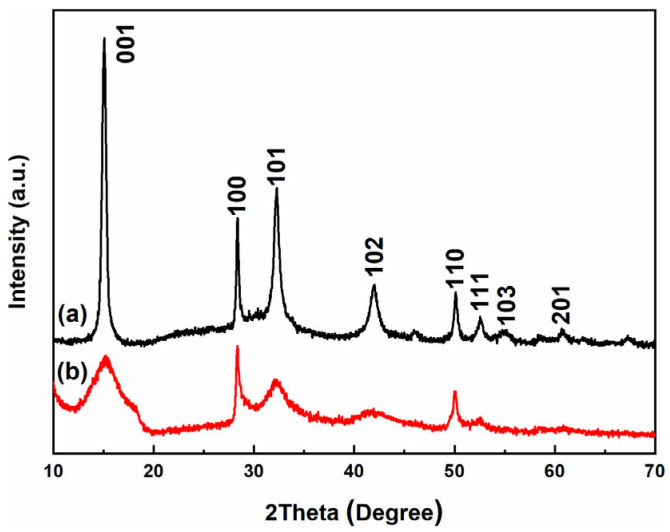
XRD patterns of nanomaterial samples from hydrothermal reaction: (a) SnS_2_ (black line); (b) Cu-SnS_2_-C (red line).

A typical XRD pattern of the as-synthesized nanomaterial samples is depicted in Figure 4 (black line) Well-defined diffraction peaks can be observed, which are indexed to the crystalline hexagonal phase of SnS_2_ in accordance with JCPDS card No. 23-0677.

Interestingly, no impurity peaks are detected, demonstrating the high phase purity of the as-prepared material. The XRD pattern of the Cu-C-SnS_2_ sample, prepared using 10 mg CuCl_2_·2H_2_O and 3 mL CDs, is displayed in Figure 4 (red line). Both samples present almost identical diffraction features characteristic of the hexagonal phase SnS_2_ (JCPDS card no. 23-0677) [24]. Comparison between the two patterns confirms that the incorporation of Cu and CDs does not destroy the hexagonal phase structure of SnS_2_.

**Figure 5:**
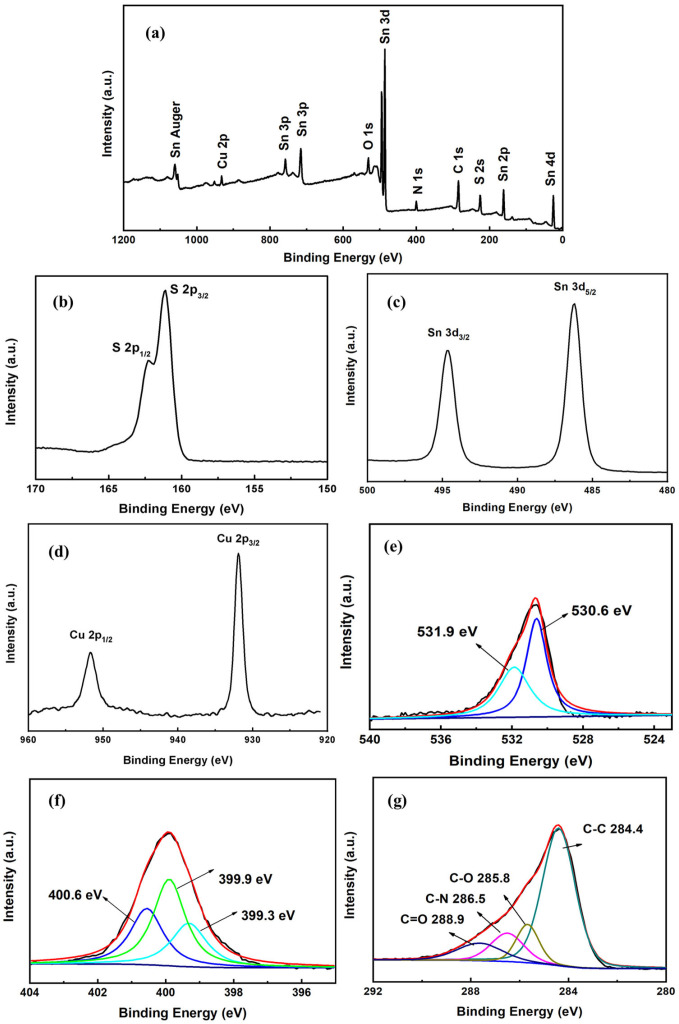
XPS spectra of Cu-C-SnS_2_ nanosheets: (**a**) survey; (**b**) S 2p; (**c**) Sn 3d; (**d**) Cu 2p; (**e**) O 1s; (**f**) N 1s; (**g**) C 1s.

The surface elemental composition and chemical valence states of the as-synthesized Cu-C-SnS_2_ nanosheets are further analyzed by means of X-ray photoelectron spectroscopy (XPS). No distinct impurity-related signals are detected in the full XPS survey spectrum, as presented in Figure 5a.

Two strong peaks located at approximately 486.6 eV and 495 eV are observed in Figure 5b, which are assigned to the Sn 3d_5_/_2_ and Sn 3d_3_/_2_ core levels, respectively. In addition, the characteristic binding energy of S 2p_3_/_2_ for the sample is detected at 161.50 eV in Figure 5c, and this value is found to be consistent with the reported data for S^2−^ species in SnS_2_ [43,44]. No signal corresponding to Sn^2+^ (with a binding energy of 485.8 eV) is identified in the obtained spectra. Only characteristic peaks assigned to Sn, S, Cu, C, N and O are detected in the spectral profile, which suggests that the content of impurity phases is below the detection resolution of XPS [45]. The high-resolution XPS spectrum corresponding to the Cu 2p orbital is presented in Figure 5d. Characteristic peaks ascribed to Cu 2p_3_/_2_ and Cu 2p_1_/_2_ are detected at approximately 932.3 eV and 952.2 eV, respectively. No distinct shake-up satellite peaks are observed in the higher binding energy range, which excludes the existence of Cu(II) species within the samples. Accordingly, copper ions in the Cu-doped SnS_2_ nanosheets are verified to be present in the +1 oxidation state. The generation of Cu^+^ is considered to be induced by the introduction of cysteine, which possesses moderate reducing properties [26]. The O 1s, N 1s and C 1s spectra are collected to confirm the introduction of the CDs in the hydrothermal reaction, and the peak positions are consistent with the reported values for CDs [42]. The high-resolution O 1s spectrum (Figure 5e) shows a characteristic peak at 530.6 eV assigned to C=O-containing functional groups, while the peak at 531.9 eV is attributed to sp^2^ C–O bonds. The high-resolution N 1s spectrum (Figure 5f) exhibits two peaks at 399.3 and 399.9 eV corresponding to pyridinic N, and the peak at 400.6 eV is associated with N–H groups. The high-resolution C 1s spectrum (Figure 5g) displays four characteristic peaks at 284.4, 285.8, 286.5 and 288.9 eV, which belong to C–C, C–O, C–N and C=O/O–C=O bonds, respectively. Additionally, XPS results confirm that morphological regulation exerts no obvious influence on the XPS spectral profiles of the samples. Collectively, these results reveal that Cu doping, combined with CD modification, effectively regulates the surface chemical environment, thereby endowing the material with enhanced optoelectronic performance.

**Figure 6:**
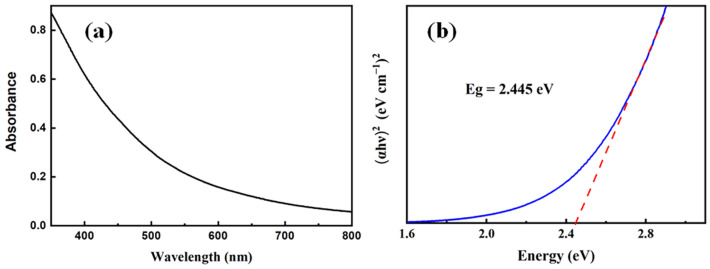
(**a**) UV–vis absorption characteristic of Cu-C-SnS_2_ nanosheets dispersed in aqueous solution. (**b**) Optical bandgap of roughly 2.445 eV is deduced for Cu-C-SnS_2_ nanosheets.

The optical property of the as-prepared Cu-C-SnS_2_ nanosheets are regarded as highly critical for their potential utilization in optoelectronic devices. The UV–vis absorption spectra of the as-prepared samples dispersed in ethanol are provided in Figure 6a.

It is revealed that efficient optical absorption is exhibited by the sample across almost the whole visible light region, which is generally recognized to span the wavelength range of 400–800 nm. The existence of such a wide response range is believed to endow the nanomaterial samples with outstanding visible-light responsiveness [33]. Furthermore, the optical bandgap values of the synthesized samples are derived on the basis of the measured absorption spectra. The plots corresponding to (*αhν*)^2^ as a function of *hν* for the prepared samples are presented in Figure 6b. The optical bandgap value can be calculated via the well-known Tauc relationship, which is expressed as follows [43]:

(1) $${\left(\alpha h\nu \right)}^{2}=B(h\nu -E_{g})$$

Among the key parameters utilized in optical analysis, *B* represents a constant, α denotes the absorption coefficient, *hν* refers to the incident photon energy, and *E*_g_ corresponds to the optical bandgap of the semiconductor material. The optical bandgap of the as-synthesized Cu-C-SnS_2_ nanosheets is determined to be 2.445 eV, which is obtained via extrapolation of the linear region in the plot of (*αhν*)^2^ against *hν* to the point where *α* equals zero. This measured value is found to be compatible with data reported in the previous literature [46].

**Figure 7:**
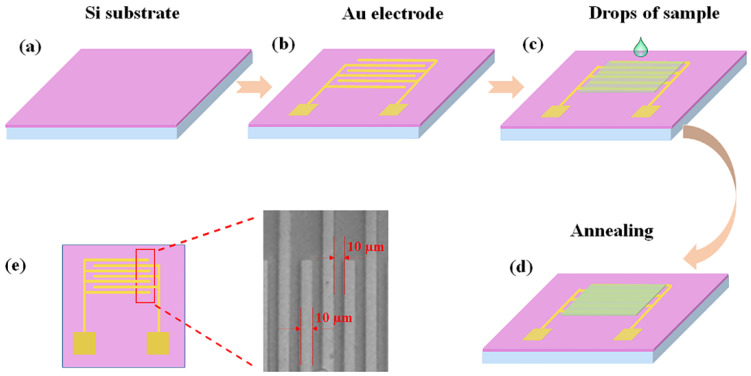
(**a**–**e**) The detailed processing steps utilized in the fabrication of Cu-C-SnS_2_ thin-film photodetectors.

To explore the photoelectric property of the as-prepared Cu-C-SnS_2_ nanosheets, a photoelectric device was fabricated. The key manufacturing processes adopted for the construction of photodetectors with the Au/Cu-C-SnS_2_/Au configuration are clearly displayed in Figure 7.

**Figure 8:**
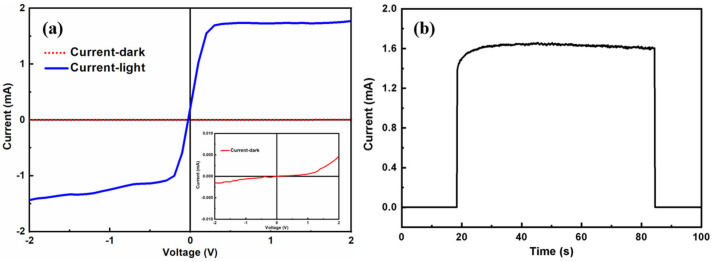
(**a**) The I–V characteristics of the photoelectronic device based on the Cu-C-SnS_2_ nanosheets, with curves recorded in darkness (red dashed line) and under 405 nm ultraviolet light (blue line). The inset shows the amplified dark current. (**b**) The corresponding I–T characteristic of the investigated device under a direct-current bias voltage of 1 V.

Commercially sourced silicon substrates were utilized as the initial supporting material (Figure 7a), and ultrasonic cleaning was performed in acetone, ethanol, and deionized water in sequence, with a duration of 5 min applied for each individual cleaning operation. Following the cleaning treatment, Au electrodes were successfully patterned on the substrates through the photolithography method. The finger-type Au contact electrodes were defined with a length of 360 μm and a width of 10 μm, and the gap between neighboring electrode fingers was precisely maintained at 10 μm, as indicated in Figure 7b. Subsequent to the aforementioned treatments, the Cu-C-SnS_2_ nanosheet thin films were prepared by dropping the Cu-C-SnS_2_ nanosheets solution (Figure 7c). The following drying treatment was performed at 100 °C for 10 min in a vacuum atmosphere, with the aims of removing the residual solvent and improving the surface coverage of the nanosheet films, as shown in Figure 7d. The digital image of the as-fabricated device constructed from Cu-C-SnS_2_ nanosheets is provided in Figure 7e. Figure 8a presents the representative current–voltage (I–V) characteristics recorded for the fabricated Cu-C-SnS_2_ nanosheet-based device within a scanning voltage window from −2 V to +2 V, with measurements conducted both in a dark environment (red dashed line) and under 405 nm ultraviolet light irradiation (blue line).

Corresponding current–time (I–T) behavior of the same device biased at a constant voltage of 1 V is illustrated in Figure 8b. A pronounced enhancement in electrical current is observed under light illumination relative to the dark condition, from which the superior photoresponse performance of the as-synthesized Cu-C-SnS_2_ nanosheets can be verified.

Notably, the device delivers a prominent photocurrent with an extremely low dark current. Compared with the pristine SnS_2_ photodetector reported by Yafei Zhang et al. [33], the Cu-C-SnS_2_ device in this work exhibits a remarkably improved photocurrent, further demonstrating its excellent potential for optoelectronic applications. Such remarkable performance improvement originates from the synergistic effect of copper doping and CD modification. This dual-modification strategy optimizes the microstructure and electronic properties of SnS_2_ nanosheets, endowing the device with superior photoresponse behavior.

## 4. Conclusions

Through a facile CD-assisted hydrothermal method, Cu-C-SnS_2_ nanosheets have been successfully synthesized. Experimental results verify that Cu dopants and CDs jointly regulate the morphological characteristics of the nanosheets, enabling the samples to become significantly thinner. Furthermore, the assembled photoelectric device exhibits an excellent photoelectric response, and an evident synergistic enhancement mechanism between Cu doping and CDs is clarified. This facile and efficient synthetic strategy provides a promising route for the controllable fabrication of SnS_2_ materials with well-tailored morphology and optimized optoelectronic performance.

## Data Availability

The original contributions presented in this study are included in the article. Further inquiries can be directed to the corresponding authors.
